# Comparison of disaster information from various media in strengthening ecological communication during & after natural disasters

**DOI:** 10.1371/journal.pone.0264089

**Published:** 2022-03-10

**Authors:** Fadhliah, Taqyuddin Bakri, Rahmat Hidayatullah, Muhammad Fardhal Pratama, Mohammad Alfit Laihi, Isrun, Muhammad Khairil, Muhammad Nur Ali, Muhammad Basir-Cyio

**Affiliations:** 1 Department of Communication Science, Faculty of Social and Political Science, Tadulako University, Palu, Indonesia; 2 Department of Languages and Arts Education, Faculty of Teacher Training and Education Science, Tadulako University, Palu, Indonesia; 3 Department of Mathematics Education, Faculty of Teacher Training and Education Science, Tadulako University, Palu, Indonesia; 4 Department of Agribusiness, Faculty of Agriculture, Tadulako University, Palu, Indonesia; 5 Department of Agroecotechnology, Faculty of Agriculture, Tadulako University, Palu, Indonesia; 6 Department of Sociology, Faculty of Social and Political Science, Tadulako University, Palu, Indonesia; National Textile University, PAKISTAN

## Abstract

This study aimed to determine the relationship between the level of panic and the various media disaster information modalities available during and after a natural disaster event. The method used was a Mix Methods Research Approach, which is a combination of qualitative descriptive and quantitative exploratory approaches. There were 150 respondents for the three research locations at Palu City, Sigi Regency and Donggala Regency. Respondents were selected by considering the event conditions experienced, physical damage to their house and their educational background. Media sources of disaster information analyzed were TV, internet, mobile phone (WA/SMS), radio, mosque/church, *surau*, community leaders and word of mouth. The data used was Likert scale analyses for perception tested with Rank Spearman Correlation. The results showed that the most significant panic level (α<0.01) was when the internet was not working, cellphones could not be used, and radio broadcasts could not be received. The most effective sources of disaster information in promoting a resilience attitude were guidance and advice from community leaders and ecological communication that was built from word of mouth. The exposure to natural disasters was shown to unite peoples’ hearts in friendship. despite some did not communicate with each other before the disaster, some were even hostile. As many as 78.6% of respondents admitted that the affection between them as victims actually appeared when natural disasters destroyed the joints of their lives, even amongst those who did not communicate with each other, or were even hostile, before the disaster. Out of ecological communication, a “strong hug due to natural disasters” was born.

## Introduction

Natural disasters that continue to hit Indonesia from year to year have increased in terms of the type of disaster, the area affected and the death toll number. Natural disasters also have an impact on loss of built property and loss of agricultural land as a source of livelihood. In the period from 2018 to 2020, there were recorded 8,645 natural disasters, in which 1,999 natural disasters occurred in 2018, 3,721 natural disasters occurred in 2019 and 2,925 natural disasters occurred in 2020. The most dominant natural disasters in those years were earthquakes, tsunamis, liquefaction, floods, flash floods, landslides, hurricanes, droughts and forest and land fires [1–3].

Central Sulawesi is one of the provinces most frequently struck by natural disasters in the past three years. Geographically, Central Sulawesi Province is located between 2°22′ North Latitude and 3°48′ South Latitude, and 119°22′ and 124°22′ East Longitude and is crossed by a major tectonic earthquake zone. The earthquake followed by tsunami and liquefaction in 2018 that hit the Palu region as is closely related to this tectonic earthquake path that crosses Central Sulawesi. In 2019, floods and landslides occurred in Sigi Regency and destroyed the two most severely affected districts, namely South Dolo District and Kulawi District.

Based on data from the Regional Disaster Management Agency (BNPBD) of Central Sulawesi, three areas have become points of concern, namely Palu City, Sigi Regency, and Donggala Regency, which were hit the most by natural disasters during 2018–2020. Natural disasters that occurred are earthquakes, tsunamis, liquefaction, flash floods, and landslides. The number of victims impacted by the natural disasters in those three years was no less than 30 thousand people, both those who died, were injured, or those who lost their property and sources of livelihood. Losses due to natural disasters within a period of three years reached tens of trillions of rupiah, and were greatly felt by the people in the three regions [4, 5].

In various natural disasters, people are often reach a state of panic. Panic is defined as the sudden onset of excessive fear or anxiety, and is characterized by a rapid heartbeat, shortness of breath, dizziness, muscle tension, or shaking [6].

One cause of panic is the disinformation related to mitigation and natural disasters [7]. In addition, limited sources of information trigger panic so resignation in facing natural disasters is more dominant [8, 9]. Victims continue to falter as a result of the unavailability of information and reliable transfer of information. The limitations of information facilities and infrastructure also exacerbate the breakdown of communication related to various important information, both during a disaster and post-disaster [10, 11].

The results of initial interviews within communities of the three regions explained that disinformation often occurs during and after natural disasters. Excessive panic hinders thinking logically and tactically about ways to save oneself, and also in not understanding how to keep one’s self safe in dealing with disasters. When people are stricken by a natural disaster, they need clarity and certainty of information, including information on events, ways to save themselves, anticipation of aftershocks and evacuation locations, through integrated communication from authorities. Unclear and uncertain information, apart from causing public panic, is also prone to being exploited by irresponsible people or parties to commit crimes.

The role of the government is to prepare and provide types and sources of information early on, especially in areas that have been routinely affected by natural disasters, such as landslides, flash floods, and earthquakes. The government has the autonomy and authority to determine, decide, and announce the status of the level of occurrence of natural disasters [12], and has suitable facilities and resources, both for use as disaster mitigation and response after natural disasters [13]. However, the wrong type and source of information will create panic and discourage the community in dealing with natural disaster situations. The anxiety and panic that had been felt by the community will be reduced and dissipated with the presence of the leadership through the timely provision of the right types and sources of information. The events in three areas in Central Sulawesi after the earthquake, tsunami and liquefaction in 2018, should be insights for the government, including the natural disasters of landslides and flash floods that hit Sigi Regency. During the disaster and after the disaster, the community was struck by panic in the form of massive exoduses and chaos in receiving aid, until looting in several places was triggered by lack of clarity in terms of control, caused by disinformation that occurred in the community.

For this reason, the government needs to prepare a tiered formulation of types and sources of information. The right formulation will make the conveyed information clear for acceptance by the public. The accuracy of the formulation also ensures that government levels share mutual information with each other, which if not done, can actually trigger public confusion. Information systems from the central government to the sub-district/village level need to be prepared for the dissemination of information along with information content that can control panic and chaos in communities affected by natural disasters. In terms of receiving information, effective media are those that use electronic means and link information from community leaders and among disaster-affected residents. The electronic means commonly used are television, radio, internet, and mobile phones, while non-electronic means are announcements from mosques/churches, surau, community leaders and word of mouth. However, in disaster conditions, electronic facilities often experience access and network problems.

The ongoing power of information between citizens is due to the plight of suffering and a sense of belonging among them and their environment which leads to strong ecological communication. In fact, while natural disasters befall the community, they cause the destruction of the connectivity of society, loss of property, loss of loved ones. But behind all these negative impacts, there is a social value that only appears and occurs when there is a terrible disaster that befalls many people. In fact, we witnessed the shared cries and strong hugs between those who have been enemies for decades, those who normally did not greet each other, nor communicate with each other. In fact, disasters that destroy the ecology actually foster a sense of affection which is indicated by the birth of the “Ecological Hug”, a hug that occurs suddenly between individuals or communities that previously closed themselves to each other. This is not a benefit of a natural disaster, but it is more accurately described as a "response in the form of affection due to a natural disaster stimulus" which is in line with the "Stimulus-Organism-Response" Theory [14, 15].

This study aimed to identify effective media in the formulation of communication for the community during natural disasters to prevent (or avoid) excessive panic in the community. The formulation of types and sources of information can be a reference for the government, especially the Central Sulawesi provincial government as one of the areas prone to natural disasters.

## Methodology

### Locations and approaches

**Fig 1:**
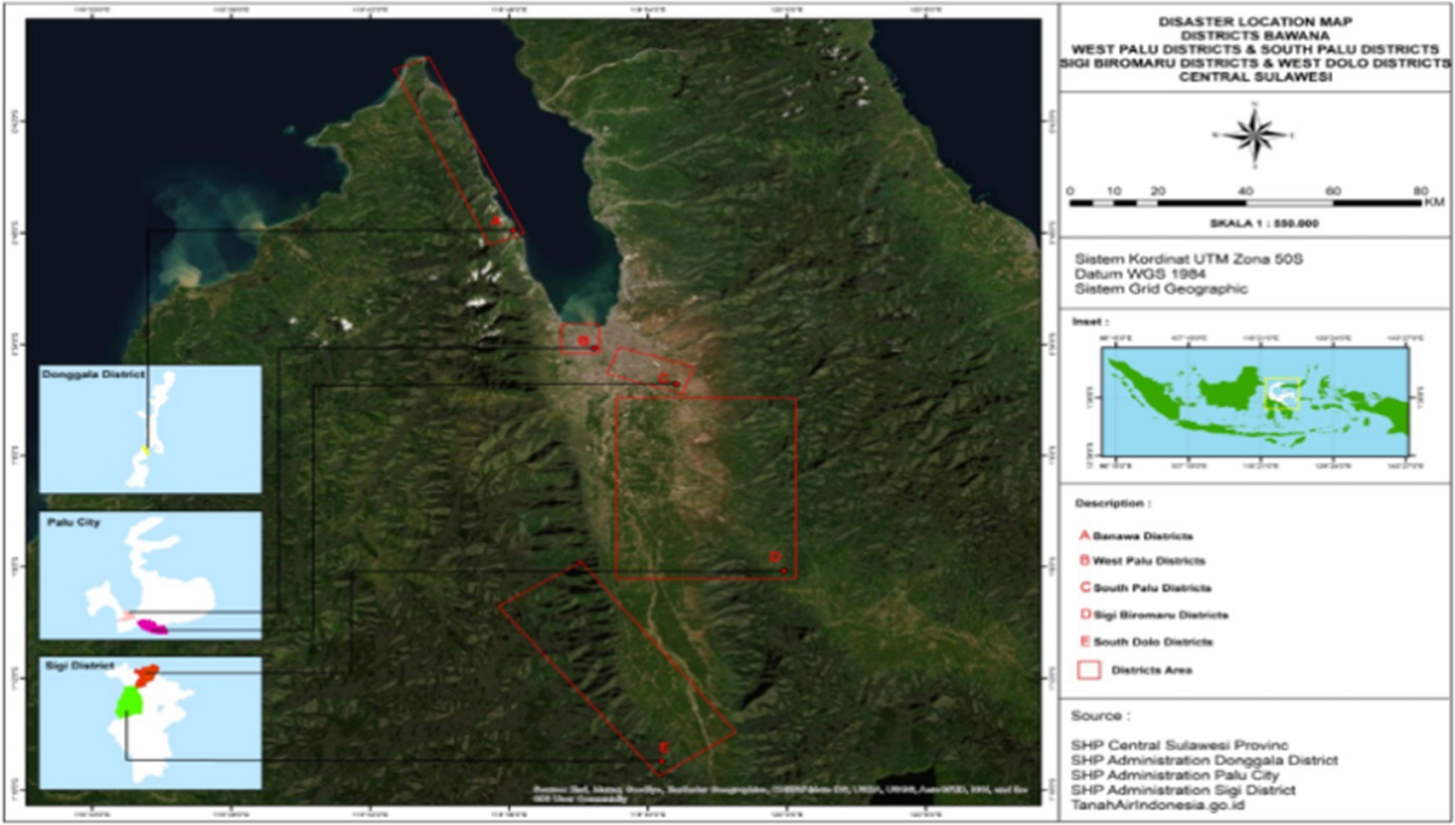
Map of research locations in Palu City, Sigi Regency & Donggala Regency.

This study used a mixed methods research approach, namely descriptive quantitative and qualitative exploratory which was carried out from August 2020 to February 2021 after Central Sulawesi was struck by various types of natural disasters from 2018 to 2020. The observation points and interviewees were determined purposively, namely Palu City, Sigi Regency and Donggala (Fig 1).

In this study, eight disaster information media variables were identified which were assumed to be a source of information for people affected by natural disasters. The eight media distinguished in the three affected areas were TV, Radio, Internet, Cellular Phones (WhatsApp/SMS), Mosques/Churches, *Surau*, Community leaders, and word of mouth. There were five categories of important participant information collected: gender, age, education level, income, and home damage.

This research was carried out under the Official Research Assignment Letter Number No: 5411/UN28/KP/2020, signed by Vice Rector for Financial and General Administration, and Ethical Clearance Number No: 0019/TU-UTD.Komisi II/UN.28/2020 from The Commission of Norms, Ethics, Feasibility, and Appropriateness of Tadulako University. Ethical Clearance was issued in accordance with the Declaration of Helsinki Ethical Principles for Medical Research involving human subjects. Consent from the community as participants were obtained, and the personal details were removed and were not published anywhere in the paper.

**Fig 2:**
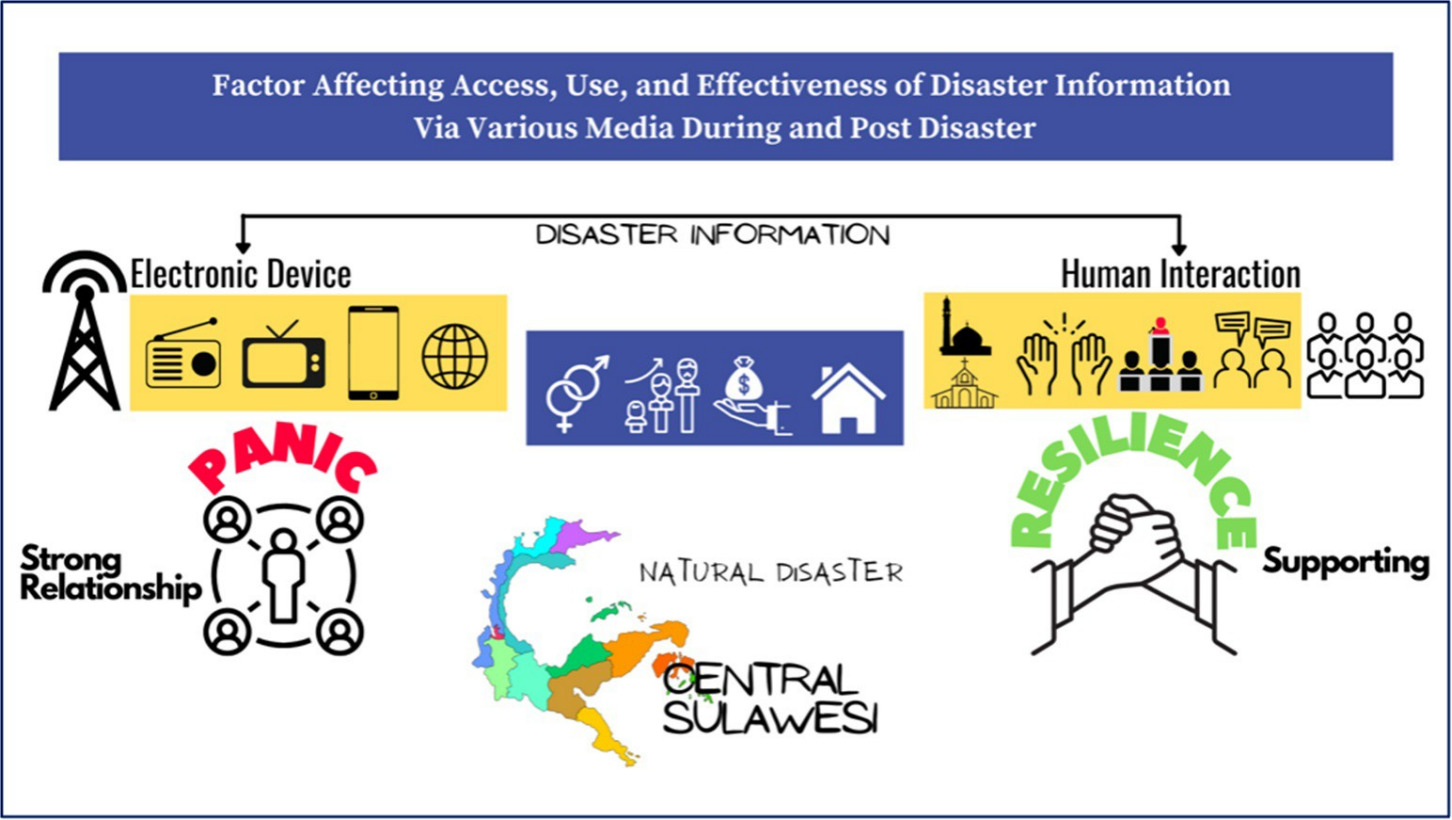
Illustration of relationship between disaster information from various media and the panic of affected community during the event of natural disaster.

The results of this research will be used to develop a natural disaster management policy, especially in strengthening communication based on electronic and human-based media. In a helpless condition, residents affected by natural disasters and environmental destruction, information and media are a source of strength in facing their lives from time to time. The media is one of the key factors in balancing panic, despair, and the desire to survive as illustrated by the relationship between the components presented in (Fig 2).

### Samples and procedures

The survey was conducted with the help of a five-person research team plus several enumerators, adjusted to the needs in the field who visited the designated observation locations and interviews. The specified locations are; (i) for Palu City, two points are determined, namely Petobo and West Palu; (ii) Sigi Regency has two points, namely Jono Oge and South Dolo; (iii) Donggala Regency is set at one point, namely Banawa District (Fig 1). Respondents who participated in the short interview were those who were affected by the disaster regardless of the type of disaster they experienced. The number of respondents for the city of Palu was 75 respondents, Sigi was 75 respondents and Donggala was 50 respondents. Data was collected for almost 6 months, August 2020 to February 2021. To strengthen the data from interviews, this study also collected data for the last one year after natural disasters. The age of the respondents ranged from 20 to 65 years. divided into two categories young (≤ 40 years) and old (> 40 years). The study samples were 53% women and 47% men.

### Measurement

Respondents were asked to provide demographic information, including: age, which was divided into two categories namely young (≤ 40 years) and old (> 40 years); their monthly income; and education divided into two variables on a scale with elementary, middle, and high school education levels in category (1), while Bachelors and above in category (2). Respondents were also interviewed about whether they received disaster information from (i) TV; (ii) Radio; (iii) Internet, (iv) Handphone (WA/SMS), (v) Mosque/church, (vi) *Surau*, (vii) community leaders and/or (viii) word of mouth. Finally, respondents were also asked questions regarding the condition of their houses, were they destroyed or still intact during a natural disaster. To determine the ease of obtaining access to disaster information from various media related to panic or resilience, measures were based on a closed questionnaire using a Likert scale. Respondents were asked to provide answers related to the level of panic experienced based on the information provided from eight types of media, both during disasters and after natural disasters. The obtained disaster management data results of the questionnaire were tested for the level of relationship using Spearman Rank Correlation Analysis with the following formula:

Spearman Rank Formula:

$$\rho =1-\frac{6\sum d_{i}^{2}}{n\left(n^{2}-1\right)}$$ (1)

Where;

*ρ* = *Correlation value of Spearman Rank*

*d*^2^ = *Difference in each pair of Rank*

n = Number of Rank Pair for Spearman

The value of the Likert scale in question is; (1) Absolutely did not panic (STP); (2) Did not panic (TP); (3) Moderate panic (CP); (4) Panic (P); and (5) Extreme panic (SP). To assess the eight types of disaster information media, a Likert scale, was also used, namely: (1) Very Easy to Access (SMM); (2) Easy access (MM); (3) Quite difficult to access (CSM); (4) Difficult to Access (SM); and, (5) Very difficult to access (SSM). The questionnaire used in the measurement was tested for reliability where the results of the reliability test are obtained by calculating the reliability using the formula "Alpha Cronbach". The results obtained are the value of "Alpha Cronbach” is more than 0.600, and validity value to ensure the questionnaire used had the ability to provide accurate results where it can be seen that all questions have valid status, because the value of r_count_ (Corrected Item-Total Correlation) > r_table_. The questionnaires distributed to the three affected areas are descriptively presented in Tables 1–3 as follows:

**Table 1:** Overall channel use patterns in Palu City.

Type of Media	% had access	% use daily	*Palu City Significant differences in frequency of channel use*				
			Sex	Age	Education	Income	Home Demage
TV	12.7	6.4	M > F	Y > O	H > L	H > L	I >D
Internet	10.2	4.7	M > F	Y > O	H > L	H > L	I > D
WA/SMS	30.9	25.6	M < F	Y > O	H > L	H > L	I < D
Radio	22.7	13.4	M > F	Y > O	H < L	H > L	I < D
Mosque/Church	13.5	2.4	M > F	Y < O	H < L	H < L	I < D
*Surau*	8.8	1.9	M > F	Y > O	H < L	H > L	I < D
Community leader	20	11.5	M > F	Y < O	H > L	H < L	I > D
Word of Mouth	77.5	60.8	M < F	Y = O	H < L	H > L	I < D
Dominant Media Users:			M>F	Y>O	H = L	H>L	I<D

Y is younger (≤ 40); O is older (> 40); H is higher education, income, or information adequacy (above the median); L is low in education, income, or information adequacy (below the median); D is destroyed house; I is intact house. Questions about the use of information sources asked specifically about SMS, while 30.9% had access to mobile phones that worked with SMS capabilities, but word of mouth was dominant (77.5%).

**Table 2:** Overall channel use patterns in Sigi Regency.

Type of Media	% had access	% use daily	*Sigi District Significant differences in frequency of channel use*				
			Sex	Age	Education	Income	Home Demage
TV	9.4	5.8	M < F	Y > O	H < L	H > L	I < D
Internet	8.3	3.4	M < F	Y > O	H > L	H > L	I < D
WA/SMS	21.3	4.8	M < F	Y > O	H < L	H > L	I < D
Radio	13.7	10.4	M > F	Y > O	H < L	H < L	I < D
Mosque/Church	12.7	6.5	M > F	Y > O	H < L	H > L	I < D
*Surau*	8.9	4.8	M > F	Y > O	H < L	H > L	I < D
Community leader	66.3	35.2	M > F	Y > O	H < L	H > L	I < D
Word of Mouth	75.6	55.7	M < F	Y< O	H < L	H < L	I < D
Dominant Media Users:			M = F	Y>O	H<L	H>L	I<D

Y is younger (≤ 40); O is older (> 40); H is higher education, income, or information adequacy (above the median); L is low in education, income, or information adequacy (below the median); D is destroyed house; I is intact house. Questions about the use of information sources asked specifically about SMS, while 21.3% had access to mobile phones that worked with SMS capabilities, but community leaders (66.3%) and word of mouth (75.6%) were dominant.

**Table 3:** Overall channel use patterns in Donggala Regency.

Type of Media	% had access	% use daily	*Donggala District Significant differences in frequency of channel use*				
			Sex	Age	Education	Income	Home Demage
TV	9.2	3.9	M < F	Y > O	H > L	H > L	I < D
Internet	12.6	7.4	M < F	Y > O	H < L	H < L	I > D
WA/SMS	30.6	14.3	M < F	Y > O	H < L	H > L	I > D
Radio	15.7	8.3	M > F	Y < O	H > L	H < L	I > D
Mosque/Church	13.5	9.6	M < F	Y < O	H < L	H < L	I > D
*Surau*	9.6	6.7	M > F	Y < O	H > L	H < L	I < D
Community leader	45.4	37.6	M > F	Y > O	H < L	H < L	I < D
Word of Mouth	56.4	45.6	M < F	Y< O	H < L	H < L	I < D
Dominant Media Users:			M<F	Y = O	H<L	H<L	I = D

Y is younger (≤ 40); O is older (> 40); H is higher education, income, or information adequacy (above the median); L is low in education, income, or information adequacy (below the median); D is destroyed house; I is intact house. Questions about the use of information sources asked specifically about SMS, while 30.6% had access to mobile phones that worked with SMS capabilities, but word of mouth was dominant (56.4%).

## Results

The results of the Spearman Rank analysis on significance (α < 0.05 & 0.01) are used to determine the relationship between disaster information from media types and the level of panic during natural disasters and after natural disasters are presented in Table 4 for each research area, namely City Palu (4A), Sigi Regency (4B) and Donggala Regency (4C).

**Table 4:** Results of correlation analysis between media types and the panic level of affected communities in Palu (A), Sigi (B) & Donggala (C).

**A. Palu City**									
Number	Variety of Media	1	2	3	4	5	6	7	8
1	Panic Level								
2	TV	.274*							
3	Internet	.872**	.161						
4	Handphone WA/SMS	.808**	.312**	.791**					
5	Radio	.547**	.250*	.404**	.382**				
6	Mosque/Church	.475**	.075	.280**	.134**	.319**			
7	*Surau*	.601**	.150	.419**	.337**	.385**	-.036		
8	Community leader	.921**	.257*	.902**	.877**	.420**	.401**	.384**	
9	Word of Mouth	.751**	.300**	.577**	.687**	.439**	.366**	.455**	.696**
*ρ < 0.05; ** ρ < 0.01. The dominant variable has a very significant correlation.									
**B. Sigi Regency**									
Number	Variety of Media	1	2	3	4	5	6	7	8
1	Panic Level								
2	TV	.275*							
3	Internet	.696**	.186						
4	Handphone WA/SMS	.604**	.200	.753**					
5	Radio	.673**	.270*	.436**	.530**				
6	Mosque/Church	.467**	.087	.253*	.134	.231*			
7	*Surau*	.549**	.192	.406**	.337**	.433**	-.036		
8	Community leader	.742**	.235*	.809**	.824**	.440**	.416**	.369**	
9	Word of Mouth	.781**	.229*	.618**	.585**	.410**	.437**	.381**	.771**
*ρ < 0.05; ** ρ < 0.01		The dominant variable has a very significant correlation							
**C. Donggala Regency**									
Number	Variety of Media	1	2	3	4	5	6	7	8
1	Panic Level								
2	TV	.332*							
3	Internet	.317*	-.079						
4	Handphone WA/SMS	.614**	.181	-.023					
5	Radio	.674**	.133	.070	.453**				
6	Mosque/Church	.631**	.166	.277	.514**	.345*			
7	*Surau*	.519**	.023	.239	.237	.281*	.014		
8	Community leader	.559**	.253	.135	.553**	.296*	.344*	.482**	
9	Word of Mouth	.783**	.306*	.150	.551**	.627**	.449**	.376**	.433**
*ρ < 0.05; ** ρ < 0.01			The dominant variable has a very significant correlation						

Based on the results of correlation test between the media and the level of panic, the levels of closeness between the variables are presented in Tables 5–7.

**Table 5:** The relationship between types of disaster information source media and panic level of affected community in Palu Regency.

Type of Media	Panic Level	Sig value	Description
TV	0.274	0.017	Weak
Internet	0.872	< 0.001	Very Strong
Handphone	0.808	< 0.001	Very Strong
Radio	0.547	< 0.001	Strong
Worship Place	0.475	< 0.001	Moderate
*Surau*	0.672	< 0.001	Strong
Community leader	0.921	< 0.001	Very Strong
Word of Mouth	0.751	< 0.001	Strong

**Table 6:** The relationship between types of disaster information source media and panic level of affected community in Sigi Regency.

Type of Media	Panic Level	Sig value	Description
TV	0.275	0.017	Weak
Internet	0.696	< 0.001	Strong
Handphone	0.604	< 0.001	Strong
Radio	0.673	< 0.001	Strong
Worship Place	0.467	< 0.001	Moderate
*Surau*	0.549	< 0.001	Strong
Community leader	0.742	< 0.001	Strong
Word of Mouth	0.781	< 0.001	Very Strong

**Table 7:** The relationship between types of disaster information source media and panic level of affected community in Donggala Regency.

Type of Media	Panic Level	Sig value	Description
TV	0.332	0.019	Moderate
Internet	0.317	0.025	Moderate
Handphone	0.614	< 0.001	Strong
Radio	0.674	< 0.001	Strong
Worship Place	0.631	< 0.001	Strong
*Surau*	0.519	< 0.001	Strong
Community leader	0.559	< 0.001	Strong
Word of Mouth	0.783	< 0.001	Very Strong

The results of the analysis of the relationship between media sources of disaster information and the level of public panic Table 5 when they need information show that only TV is weak. This means that if residents affected by natural disasters do not receive information from TV, residents do not feel panicked. In contrast, if residents do not receive disaster information from other media, especially the internet media, cellphones, and also community leaders, they are confused and even panicked.

From the data in Table 6, the relationship between media access and the level of panic shows that TV also has a weak relationship. In the sense that if the affected people do not receive information from TV, the residents are not affected but if they do not get information from the other seven media, then they tend to panic, especially if they are not able to share information by word of mouth, including if they do not get disaster-related information through mobile phone media and from community leaders related to disaster management.

The results of the Spearman rank correlation test in Table 7 show that the information expected from community leaders, cellphones, and the internet has a strong relationship in influencing panic if the media cannot be accessed and respondents (or media outlets?) do not obtain disaster information. This means that the more difficult it is for people affected by natural disasters to obtain information from the three types of media, the more their panic will increase or their resilience in dealing with the surrounding situation will decrease. Even a strong local society if controlled by panic, then will become weak. They are of the view that the disaster information obtained from the three media, apart from being more accurate because of those who have the authority, intelligence, and concern in providing disaster information, provide certainty.

## Discussion

The results of the research into information received in both Palu City, Sigi Regency and Donggala Regency, emphasize the importance of communicating information that can provide psychosocial benefits to communities affected by natural disasters, assuring them at least to know there is a way through the next threat of the dynamics of natural disasters. The intended benefit is to the community and the environment where the community lives or where they are temporarily accommodated. Information from community leaders who have been role models so far, has the meaning of a strong ecological communication dimension associated with greater perceptions of ownership of the environment in which they live [16, 17]. The perception of environmental ownership under the leadership of local community leaders has an emotional connection that individuals have with other communities who are equally affected by natural disasters. Perception and sense of belonging to the environment, contribute to people’s lives by influencing commitment and involvement in solving problems in disaster-affected areas [18, 19].

With regard to the disaster felt by the affected communities, in Palu, Sigi and Donggala, each community reported similar perceptions as to the importance of receiving the information needed. Any information that comes from the media that has an influence, especially from SMS, community leaders and radio, becomes the basis of strength in transmitting any information content by word of mouth [20]. The communication that is built is a reflection of ecological communication between individuals so that they can minimize panic [21], as well as strengthen their resilience during natural disasters and design plans for next steps after natural disasters [22–24].

Where residents actively communicate to help their families, friends, and neighbors in overcoming each phase of a disaster, it is likely that each individual will feel something bigger than the natural disaster that befell him [25, 26]. In addition, individuals who feel part of a community can build stronger disaster ecology communication to help themselves, their families, and neighbors in overcoming the difficulties that occur as a result of the disaster [27, 28].

Likewise, information obtained by word of mouth, *surau* and radio has a strong relationship in influencing the level of panic in the community, even though there are differences between regions.

The desire to gaint access to information is often hampered by an unstable internet network. Sigi and Donggala regencies have sociological similarities which are more likely to feel the benefits of ecological communication built from community leaders, while for Palu City, electronic media is more dominant as a source of disaster information. In strengthening community resilience when dealing with natural disasters, it is closely related to communication between those who are suffering, those whose homes have been destroyed or who are still intact, or who are in temporary shelters [29, 30]. The suffering they feel when they are affected by both a disaster and physical damage to their homes and environment due to exposure to natural disasters will build more intensive individual disaster communication because they directly experience panic, stress and depression conditions related to natural disasters [31, 32]. In addition, greater exposure to disaster may also be associated with more challenging mental health reactions [33], which in turn results in post-event disaster communication so that psychic bonding of feelings is very strong [34]. Thus, exposure to natural disasters can create a context for communication actions in which people more often communicate with other individuals at the micro level of disaster communication ecology in order to overcome the challenges resulting from the disaster, including sharing their suffering with each other [35, 36].

**Fig 3:**
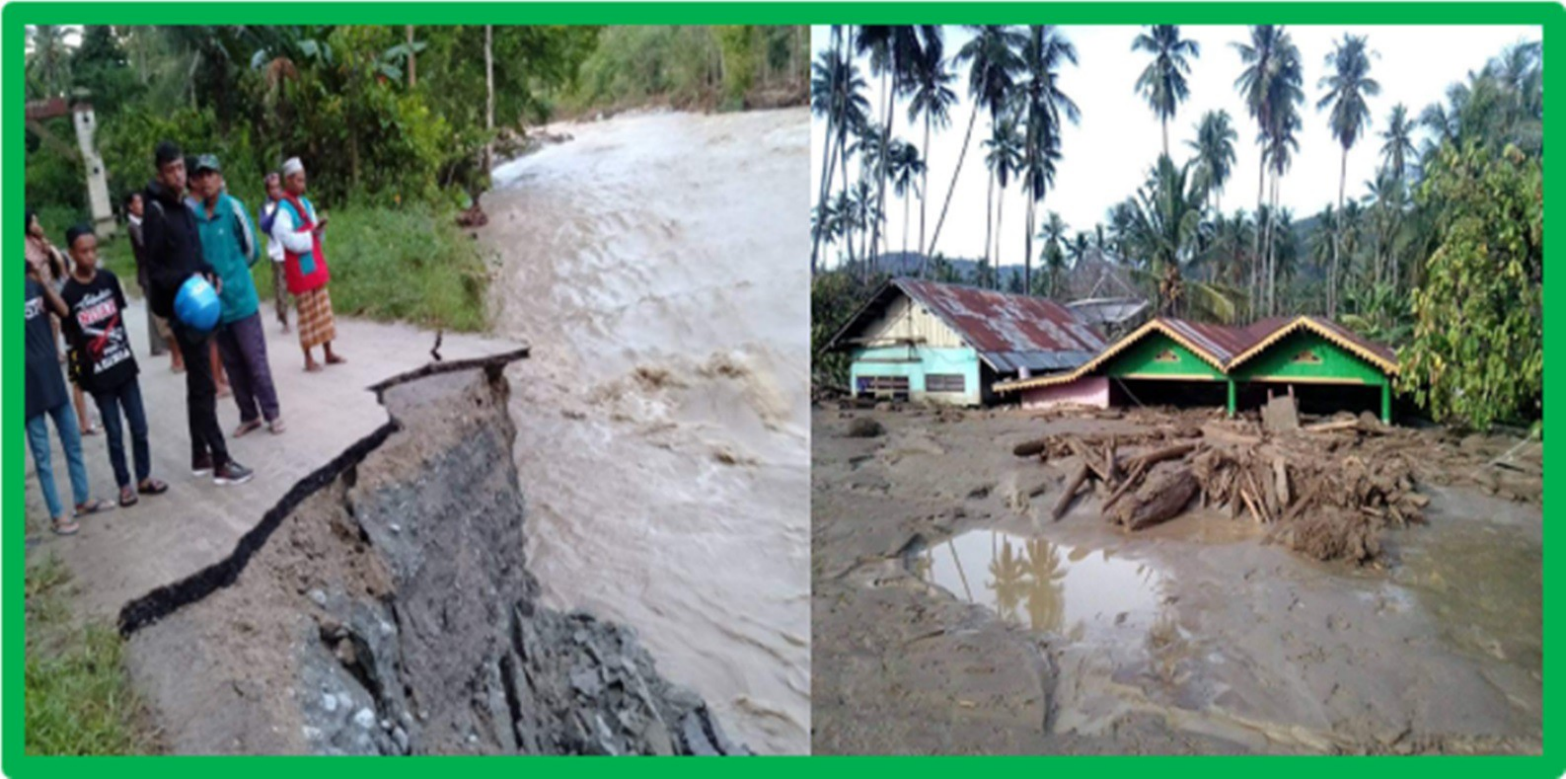
Sharing information by word of mouth attended by community leaders to give encouragement to face the flash flood in Sigi.

The facts show that word of mouth among individuals is stronger and higher, both while still in temporary shelters and after those who are exposed slowly rebuild their new lives after exposure to natural disasters, be it earthquakes, liquefaction, or landslides destroying their environment and settlements. The following illustration proves that ecological communication through word of mouth is more dominant, especially if it is accompanied by the presence of community leaders among them, as presented in (Fig 3).

Looking at the condition of the impact of natural disasters in the area where the research is located, it proves that the information media for every affected citizen applies to a micro area. The Hypodermic Needle Model [37] in communication does not apply and is ineffective during a disaster. The Syringe Model is a one-step flow from the mass media directly to the audience as the mass audience [38]. This model is also more appropriate if the socio-economic conditions of the community are in a normal atmosphere, similar to the Stimulus-Response theory [39], but when an extraordinary situation occurs due to natural disasters, ecological communication is seen as more effective [40]. In situations of natural disasters, it is clear that the Mass Audience is not known as a society that is considered as atoms that are separated from each other, not interconnected, and only associated with the mass media [41]. Sociologically, disaster-affected communities actually form small communities that are united by feelings and suffering. At the timeof panicking, panic can be a cohesive factor in strengthening individual communication and ecological communication [42]. We will not find powerful mass media in small communities that are in bondage due to natural disasters [43, 44], but what we see is cohesiveness, maintaining relationships, protecting each other, and helping each other [45, 46], who were born from the dimension of togetherness, not because of social engineering and not political engineering, but because of natural disasters. So, natural disasters can actually unite individuals or communities who are dispersed into one in a big umbrella called the "bond of fate and suffering". All the interview points generally acknowledged that some of them were neighbors who did not greet each other for decades, did not know each other, never helped each other, yet the disaster that united them was in conditions that were equally worrisome, sad, and also touching. The residents of Sigi who were interviewed said that they had disagreements with their neighbors because of the boundaries of their yards, the impact of which was passed down from generation to generation to their children. However, when the natural disaster of flash floods and earthquakes shook Sigi, they turned instantly into individuals who longed for togetherness, helping each other, and they hugged each other tightly. This strong hug is not because they miss each other but are united in their hearts because of a natural disaster, and this is what is called the "Calamity Hug" which results in "ecological communication". Changes in human attitudes are sometimes not easy in a normal atmosphere, but everything can change with natural disasters that destroy the joints of life, which at the same time become a stimulus in uniting communities because they feel suffering. 78.6% of respondents said that the growth of ecological communication and the emergence of hugs among victims were acknowledged by victims of together being exposed to natural disasters.

## Conclusion

There are several research results that can be concluded as follows:

1. In the event of a natural disaster, the affected residents really need information from various sources and media so that their panic does not last long. The most needed information is, fast assistance, shelter, and logistics.
2. Electronic information is needed because it is very helpful, but in a disaster situation, the network is often completely off or unstable. In such conditions, the presence of community leaders is needed to calm the panic of the residents. Community leaders are also expected to be a medium for connecting information to the government to convey the conditions and situations around the disaster area.
3. Disasters on the one hand are the cause of damage and destruction of various facilities, the occurrence of victims in families, but on the other hand, natural disasters have caused compassion and have been recognized by 78.6% of respondents from the total number of victims interviewed, who before the disaster were among those who were hostile, did not communicate with each other, even hated each other. Natural disasters unite their hearts, strengthen the embrace of disaster through ecological communication among victims.

## Supporting information

S1 Data Data of correlation. (XLSX)

S2 Data Data of overall channel use patterns. (XLSX)

S3 Data Data processing output. (DOCX)
