# Supplementary material for: Comparison of disaster information from various media in strengthening ecological communication during & after natural disasters
Source: PLoS One. 2022 Mar 10;17(3):e0264089. doi: 10.1371/journal.pone.0264089 (PMC8912905; doi:10.1371/journal.pone.0264089)
Supplement: S3 Data — (DOCX) [file pone.0264089.s003.docx]

Kota Palu

| **Correlations** | | | | | | | | | | | |
| --- | --- | --- | --- | --- | --- | --- | --- | --- | --- | --- | --- |
|  | | | Tingkat Kepanikan | TV | Internet | Telepoh dan SMS | Radio | Tempat Ibadah | Surau | Toko Masyarakat | Word to Mouth |
| Spearman's rho | Tingkat Kepanikan | Correlation Coefficient | 1,000 | ,274^*^ | ,872^**^ | ,808^**^ | ,547^**^ | ,475^**^ | ,601^**^ | ,921^**^ | ,751^**^ |
|  |  | Sig. (2-tailed) | . | ,017 | ,000 | ,000 | ,000 | ,000 | ,000 | ,000 | ,000 |
|  |  | N | 75 | 75 | 75 | 75 | 75 | 75 | 75 | 75 | 75 |
|  | TV | Correlation Coefficient | ,274^*^ | 1,000 | ,161 | ,312^**^ | ,250^*^ | ,075 | ,150 | ,257^*^ | ,300^**^ |
|  |  | Sig. (2-tailed) | ,017 | . | ,168 | ,006 | ,031 | ,524 | ,200 | ,026 | ,009 |
|  |  | N | 75 | 75 | 75 | 75 | 75 | 75 | 75 | 75 | 75 |
|  | Internet | Correlation Coefficient | ,872^**^ | ,161 | 1,000 | ,791^**^ | ,404^**^ | ,280^*^ | ,419^**^ | ,902^**^ | ,577^**^ |
|  |  | Sig. (2-tailed) | ,000 | ,168 | . | ,000 | ,000 | ,015 | ,000 | ,000 | ,000 |
|  |  | N | 75 | 75 | 75 | 75 | 75 | 75 | 75 | 75 | 75 |
|  | Telepoh dan SMS | Correlation Coefficient | ,808^**^ | ,312^**^ | ,791^**^ | 1,000 | ,382^**^ | ,134 | ,337^**^ | ,877^**^ | ,687^**^ |
|  |  | Sig. (2-tailed) | ,000 | ,006 | ,000 | . | ,001 | ,252 | ,003 | ,000 | ,000 |
|  |  | N | 75 | 75 | 75 | 75 | 75 | 75 | 75 | 75 | 75 |
|  | Radio | Correlation Coefficient | ,547^**^ | ,250^*^ | ,404^**^ | ,382^**^ | 1,000 | ,319^**^ | ,385^**^ | ,420^**^ | ,439^**^ |
|  |  | Sig. (2-tailed) | ,000 | ,031 | ,000 | ,001 | . | ,005 | ,001 | ,000 | ,000 |
|  |  | N | 75 | 75 | 75 | 75 | 75 | 75 | 75 | 75 | 75 |
|  | Tempat Ibadah | Correlation Coefficient | ,475^**^ | ,075 | ,280^*^ | ,134 | ,319^**^ | 1,000 | -,036 | ,401^**^ | ,366^**^ |
|  |  | Sig. (2-tailed) | ,000 | ,524 | ,015 | ,252 | ,005 | . | ,761 | ,000 | ,001 |
|  |  | N | 75 | 75 | 75 | 75 | 75 | 75 | 75 | 75 | 75 |
|  | Surau | Correlation Coefficient | ,601^**^ | ,150 | ,419^**^ | ,337^**^ | ,385^**^ | -,036 | 1,000 | ,384^**^ | ,455^**^ |
|  |  | Sig. (2-tailed) | ,000 | ,200 | ,000 | ,003 | ,001 | ,761 | . | ,001 | ,000 |
|  |  | N | 75 | 75 | 75 | 75 | 75 | 75 | 75 | 75 | 75 |
|  | Toko Masyarakat | Correlation Coefficient | ,921^**^ | ,257^*^ | ,902^**^ | ,877^**^ | ,420^**^ | ,401^**^ | ,384^**^ | 1,000 | ,696^**^ |
|  |  | Sig. (2-tailed) | ,000 | ,026 | ,000 | ,000 | ,000 | ,000 | ,001 | . | ,000 |
|  |  | N | 75 | 75 | 75 | 75 | 75 | 75 | 75 | 75 | 75 |
|  | Word to Mouth | Correlation Coefficient | ,751^**^ | ,300^**^ | ,577^**^ | ,687^**^ | ,439^**^ | ,366^**^ | ,455^**^ | ,696^**^ | 1,000 |
|  |  | Sig. (2-tailed) | ,000 | ,009 | ,000 | ,000 | ,000 | ,001 | ,000 | ,000 | . |
|  |  | N | 75 | 75 | 75 | 75 | 75 | 75 | 75 | 75 | 75 |
| *. Correlation is significant at the 0.05 level (2-tailed). | | | | | | | | | | | |
| **. Correlation is significant at the 0.01 level (2-tailed). | | | | | | | | | | | |

Kabupaten Sigi

| **Correlations** | | | | | | | | | | | |
| --- | --- | --- | --- | --- | --- | --- | --- | --- | --- | --- | --- |
|  | | | Tingkat Kepanikan | TV | Internet | Telepoh dan SMS | Radio | Tempat Ibadah | Surau | Toko Masyarakat | Word to Mouth |
| Spearman's rho | Tingkat Kepanikan | Correlation Coefficient | 1,000 | ,275^*^ | ,696^**^ | ,604^**^ | ,673^**^ | ,467^**^ | ,549^**^ | ,742^**^ | ,781^**^ |
|  |  | Sig. (2-tailed) | . | ,017 | ,000 | ,000 | ,000 | ,000 | ,000 | ,000 | ,000 |
|  |  | N | 75 | 75 | 75 | 75 | 75 | 75 | 75 | 75 | 75 |
|  | TV | Correlation Coefficient | ,275^*^ | 1,000 | ,186 | ,200 | ,270^*^ | ,087 | ,192 | ,235^*^ | ,229^*^ |
|  |  | Sig. (2-tailed) | ,017 | . | ,110 | ,086 | ,019 | ,457 | ,099 | ,043 | ,048 |
|  |  | N | 75 | 75 | 75 | 75 | 75 | 75 | 75 | 75 | 75 |
|  | Internet | Correlation Coefficient | ,696^**^ | ,186 | 1,000 | ,753^**^ | ,436^**^ | ,253^*^ | ,406^**^ | ,809^**^ | ,618^**^ |
|  |  | Sig. (2-tailed) | ,000 | ,110 | . | ,000 | ,000 | ,028 | ,000 | ,000 | ,000 |
|  |  | N | 75 | 75 | 75 | 75 | 75 | 75 | 75 | 75 | 75 |
|  | Telepoh dan SMS | Correlation Coefficient | ,604^**^ | ,200 | ,753^**^ | 1,000 | ,530^**^ | ,134 | ,337^**^ | ,824^**^ | ,585^**^ |
|  |  | Sig. (2-tailed) | ,000 | ,086 | ,000 | . | ,000 | ,252 | ,003 | ,000 | ,000 |
|  |  | N | 75 | 75 | 75 | 75 | 75 | 75 | 75 | 75 | 75 |
|  | Radio | Correlation Coefficient | ,673^**^ | ,270^*^ | ,436^**^ | ,530^**^ | 1,000 | ,231^*^ | ,433^**^ | ,440^**^ | ,410^**^ |
|  |  | Sig. (2-tailed) | ,000 | ,019 | ,000 | ,000 | . | ,046 | ,000 | ,000 | ,000 |
|  |  | N | 75 | 75 | 75 | 75 | 75 | 75 | 75 | 75 | 75 |
|  | Tempat Ibadah | Correlation Coefficient | ,467^**^ | ,087 | ,253^*^ | ,134 | ,231^*^ | 1,000 | -,036 | ,416^**^ | ,437^**^ |
|  |  | Sig. (2-tailed) | ,000 | ,457 | ,028 | ,252 | ,046 | . | ,761 | ,000 | ,000 |
|  |  | N | 75 | 75 | 75 | 75 | 75 | 75 | 75 | 75 | 75 |
|  | Surau | Correlation Coefficient | ,549^**^ | ,192 | ,406^**^ | ,337^**^ | ,433^**^ | -,036 | 1,000 | ,369^**^ | ,381^**^ |
|  |  | Sig. (2-tailed) | ,000 | ,099 | ,000 | ,003 | ,000 | ,761 | . | ,001 | ,001 |
|  |  | N | 75 | 75 | 75 | 75 | 75 | 75 | 75 | 75 | 75 |
|  | Toko Masyarakat | Correlation Coefficient | ,742^**^ | ,235^*^ | ,809^**^ | ,824^**^ | ,440^**^ | ,416^**^ | ,369^**^ | 1,000 | ,771^**^ |
|  |  | Sig. (2-tailed) | ,000 | ,043 | ,000 | ,000 | ,000 | ,000 | ,001 | . | ,000 |
|  |  | N | 75 | 75 | 75 | 75 | 75 | 75 | 75 | 75 | 75 |
|  | Word to Mouth | Correlation Coefficient | ,781^**^ | ,229^*^ | ,618^**^ | ,585^**^ | ,410^**^ | ,437^**^ | ,381^**^ | ,771^**^ | 1,000 |
|  |  | Sig. (2-tailed) | ,000 | ,048 | ,000 | ,000 | ,000 | ,000 | ,001 | ,000 | . |
|  |  | N | 75 | 75 | 75 | 75 | 75 | 75 | 75 | 75 | 75 |
| *. Correlation is significant at the 0.05 level (2-tailed). | | | | | | | | | | | |
| **. Correlation is significant at the 0.01 level (2-tailed). | | | | | | | | | | | |

Kabupaten Donggala

| **Correlations** | | | | | | | | | | | |
| --- | --- | --- | --- | --- | --- | --- | --- | --- | --- | --- | --- |
|  | | | Tingkat Kepanikan | TV | Internet | Telepoh dan SMS | Radio | Tempat Ibadah | Surau | Toko Masyarakat | Word to Mouth |
| Spearman's rho | Tingkat Kepanikan | Correlation Coefficient | 1,000 | ,332^*^ | ,317^*^ | ,614^**^ | ,674^**^ | ,631^**^ | ,519^**^ | ,559^**^ | ,783^**^ |
|  |  | Sig. (2-tailed) | . | ,019 | ,025 | ,000 | ,000 | ,000 | ,000 | ,000 | ,000 |
|  |  | N | 50 | 50 | 50 | 50 | 50 | 50 | 50 | 50 | 50 |
|  | TV | Correlation Coefficient | ,332^*^ | 1,000 | -,079 | ,181 | ,133 | ,166 | ,023 | ,253 | ,306^*^ |
|  |  | Sig. (2-tailed) | ,019 | . | ,586 | ,208 | ,358 | ,250 | ,872 | ,077 | ,031 |
|  |  | N | 50 | 50 | 50 | 50 | 50 | 50 | 50 | 50 | 50 |
|  | Internet | Correlation Coefficient | ,317^*^ | -,079 | 1,000 | -,023 | ,070 | ,277 | ,239 | ,135 | ,150 |
|  |  | Sig. (2-tailed) | ,025 | ,586 | . | ,875 | ,631 | ,052 | ,095 | ,350 | ,298 |
|  |  | N | 50 | 50 | 50 | 50 | 50 | 50 | 50 | 50 | 50 |
|  | Telepoh dan SMS | Correlation Coefficient | ,614^**^ | ,181 | -,023 | 1,000 | ,453^**^ | ,514^**^ | ,237 | ,553^**^ | ,551^**^ |
|  |  | Sig. (2-tailed) | ,000 | ,208 | ,875 | . | ,001 | ,000 | ,098 | ,000 | ,000 |
|  |  | N | 50 | 50 | 50 | 50 | 50 | 50 | 50 | 50 | 50 |
|  | Radio | Correlation Coefficient | ,674^**^ | ,133 | ,070 | ,453^**^ | 1,000 | ,345^*^ | ,281^*^ | ,296^*^ | ,627^**^ |
|  |  | Sig. (2-tailed) | ,000 | ,358 | ,631 | ,001 | . | ,014 | ,048 | ,037 | ,000 |
|  |  | N | 50 | 50 | 50 | 50 | 50 | 50 | 50 | 50 | 50 |
|  | Tempat Ibadah | Correlation Coefficient | ,631^**^ | ,166 | ,277 | ,514^**^ | ,345^*^ | 1,000 | ,014 | ,344^*^ | ,449^**^ |
|  |  | Sig. (2-tailed) | ,000 | ,250 | ,052 | ,000 | ,014 | . | ,922 | ,015 | ,001 |
|  |  | N | 50 | 50 | 50 | 50 | 50 | 50 | 50 | 50 | 50 |
|  | Surau | Correlation Coefficient | ,519^**^ | ,023 | ,239 | ,237 | ,281^*^ | ,014 | 1,000 | ,482^**^ | ,376^**^ |
|  |  | Sig. (2-tailed) | ,000 | ,872 | ,095 | ,098 | ,048 | ,922 | . | ,000 | ,007 |
|  |  | N | 50 | 50 | 50 | 50 | 50 | 50 | 50 | 50 | 50 |
|  | Toko Masyarakat | Correlation Coefficient | ,559^**^ | ,253 | ,135 | ,553^**^ | ,296^*^ | ,344^*^ | ,482^**^ | 1,000 | ,433^**^ |
|  |  | Sig. (2-tailed) | ,000 | ,077 | ,350 | ,000 | ,037 | ,015 | ,000 | . | ,002 |
|  |  | N | 50 | 50 | 50 | 50 | 50 | 50 | 50 | 50 | 50 |
|  | Word to Mouth | Correlation Coefficient | ,783^**^ | ,306^*^ | ,150 | ,551^**^ | ,627^**^ | ,449^**^ | ,376^**^ | ,433^**^ | 1,000 |
|  |  | Sig. (2-tailed) | ,000 | ,031 | ,298 | ,000 | ,000 | ,001 | ,007 | ,002 | . |
|  |  | N | 50 | 50 | 50 | 50 | 50 | 50 | 50 | 50 | 50 |
| *. Correlation is significant at the 0.05 level (2-tailed). | | | | | | | | | | | |
| **. Correlation is significant at the 0.01 level (2-tailed). | | | | | | | | | | | |
